# Case report: Innovative treatment for one metastatic thyroid-like follicular carcinoma of the kidney with *ATM* and *POLE* mutations

**DOI:** 10.3389/fonc.2024.1352865

**Published:** 2024-06-12

**Authors:** Jin-Ju Lei, Jie Rao, Hong-Yan Feng, De-Dong Cao, Hong-Lin Yan, Jing-Ping Yuan, Zhen-Min Jiang, Yi-Qiao Zhang

**Affiliations:** ^1^ Cancer Center, Renmin Hospital of Wuhan University, Wuhan, Hubei, China; ^2^ Department of Pathology, Renmin Hospital of Wuhan University, Wuhan, Hubei, China; ^3^ Department of PET/CT Center, Renmin Hospital of Wuhan University, Wuhan, Hubei, China

**Keywords:** metastatic thyroid-like follicular carcinoma of the kidney, ATM and POLE mutations, programmed death 1 receptor inhibitor, pazopanib, radiotherapy

## Abstract

Thyroid-like follicular renal cell carcinoma (TLFRCC), also known as thyroid-like follicular carcinoma of the kidney or thyroid follicular carcinoma like renal tumor, is an exceedingly rare variant of renal cell carcinoma that has only recently been acknowledged. This neoplasm exhibits a distinct follicular morphology resembling that of the thyroid gland. Immunohistochemical analysis reveals positive expression of PAX8, Vimentin, and EMA, while thyroid-specific markers TG and TTF1 are consistently absent. Furthermore, there is a notable absence of any concurrent thyroid pathology on clinical evaluation. Previous reports have suggested that TLFRCC is an indolent, slow-growing malignancy with infrequent metastatic potential. In this report, we present a case of TLFRCC characterized by remarkable ossification and widespread metastasis, including multifocal pulmonary lesions, involvement of the abdominal wall, and infiltration into the psoas muscle. To our knowledge, this represents only the third documented instance of distant metastasis in thyroid follicular renal carcinoma. The current case demonstrates a therapeutic approach that combines radiotherapy with the utilization of toripalimab, a programmed cell death 1 (PD-1) receptor inhibitor, and pazopanib. This treatment regimen was tailored based on comprehensive genomic profiling, which identified mutations in the POLE (catalytic subunit of DNA polymerase epsilon) and ATM (ataxia-telangiectasia mutated) genes, both of which have been implicated in the pathogenesis of various malignant tumors. These findings represent a novel discovery, as such mutations have never been reported in association with TLFRCC. Thus far, this therapeutic approach has proven to be the most efficacious option for treating metastatic TLFRCC among previously reported, and it also marks the first mention of the potential benefits of radiotherapy in managing this particular subtype of renal cell carcinoma.

## 1 Introduction

Thyroid-like follicular renal cell carcinoma, also known as thyroid-like follicular carcinoma of the kidney or thyroid follicular carcinoma-like renal tumor, is a rare subtype of renal cell carcinoma that was first reported by Angell et al. in 1996 and has been newly recognized in recent years (1). Studies have found that the tumor exhibits a characteristic thyroid follicular-like structure. In most cases, there is a positive expression of PAX8, vimentin, and EMA, but a negative expression for the thyroid-specific markers thyroglobulin and thyroid transcription factor-1. Additionally, no thyroid-related lesions were found during clinical examination in patients with these tumors (2–5). This tumor has unique histomorphology, immunophenotype, and clinical features, and has only been reported in a few dozen cases worldwide (6). The 2012 renal tumor consensus meeting of the International Society of Urological Pathology provided a detailed description of it (7). In The World Health Organization Classification of renal tumors (2016), it was only listed as a tentative subtype of renal cell carcinoma (8), However, in The World Health Organization 2022 classification of urinary and male genital tumors (5th edition), thyroid-like follicular renal cell carcinoma was included under “other renal tumors”, which includes a group of diverse, unrelated renal tumors that do not fit into other categories (9, 10). Previous reports have indicated that thyroid-like follicular renal cell carcinoma is an inert, slow-growing, and rarely metastasizing malignant tumor. Surgical resection, whether radical or partial excision, is nearly the sole treatment option for this malignant tumor (2, 4, 11). In this study, we report a case of Thyroid-like follicular renal cell carcinoma (TLFRCC) with significant ossification and widespread metastasis, including multiple lung lesions in both lungs, abdominal wall, and the psoas muscle. To date, this is the third reported case of TLFRCC with distant metastasis worldwide. This report presents a case of TLFRCC combined with radiotherapy, programmed death 1 receptor inhibitor toripalimab, and pazopanib. These treatments were offered based on comprehensive genomic profiling, which identified *POLE* and *ATM* mutations associated with many malignant tumors, a finding that has not been previously reported. Up to now, it’s the most effective treatment option among previously reported for metastatic TLFRCC and the first mention of the efficacy of radiotherapy in TLFRCC.

## 2 Case description

**Figure 1:**
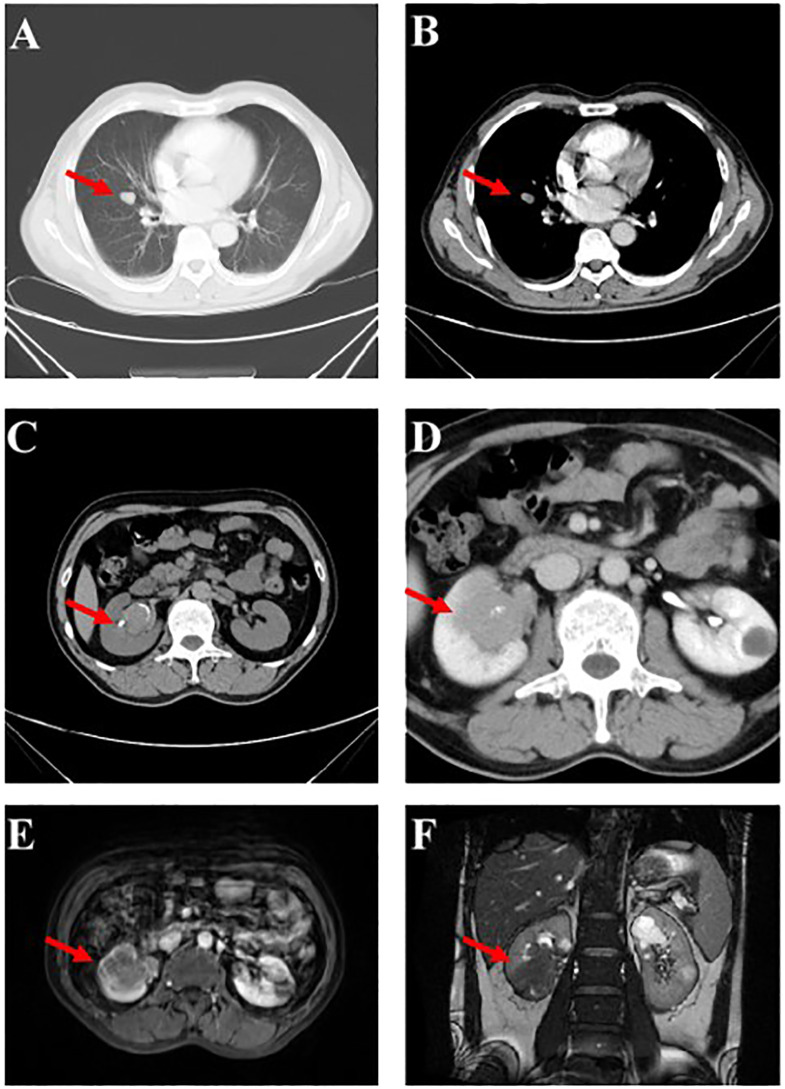
Imaging findings upon Initial Diagnosis. **(A, B)** An array of minuscule pulmonary nodules (marked by the red arrows) aroused suspicions of metastasis within the lungs. **(C, D)** Computed Tomography (CT) scans, alongside Magnetic Resonance Imaging (MRI) **(E, F)**, revealed the encroachment of a mass (marked by the red arrows) measuring 4.2 x 4.0 x 3.5cm at the inferior pole of the right kidney, encompassing the renal pelvis. .

**Figure 2:**
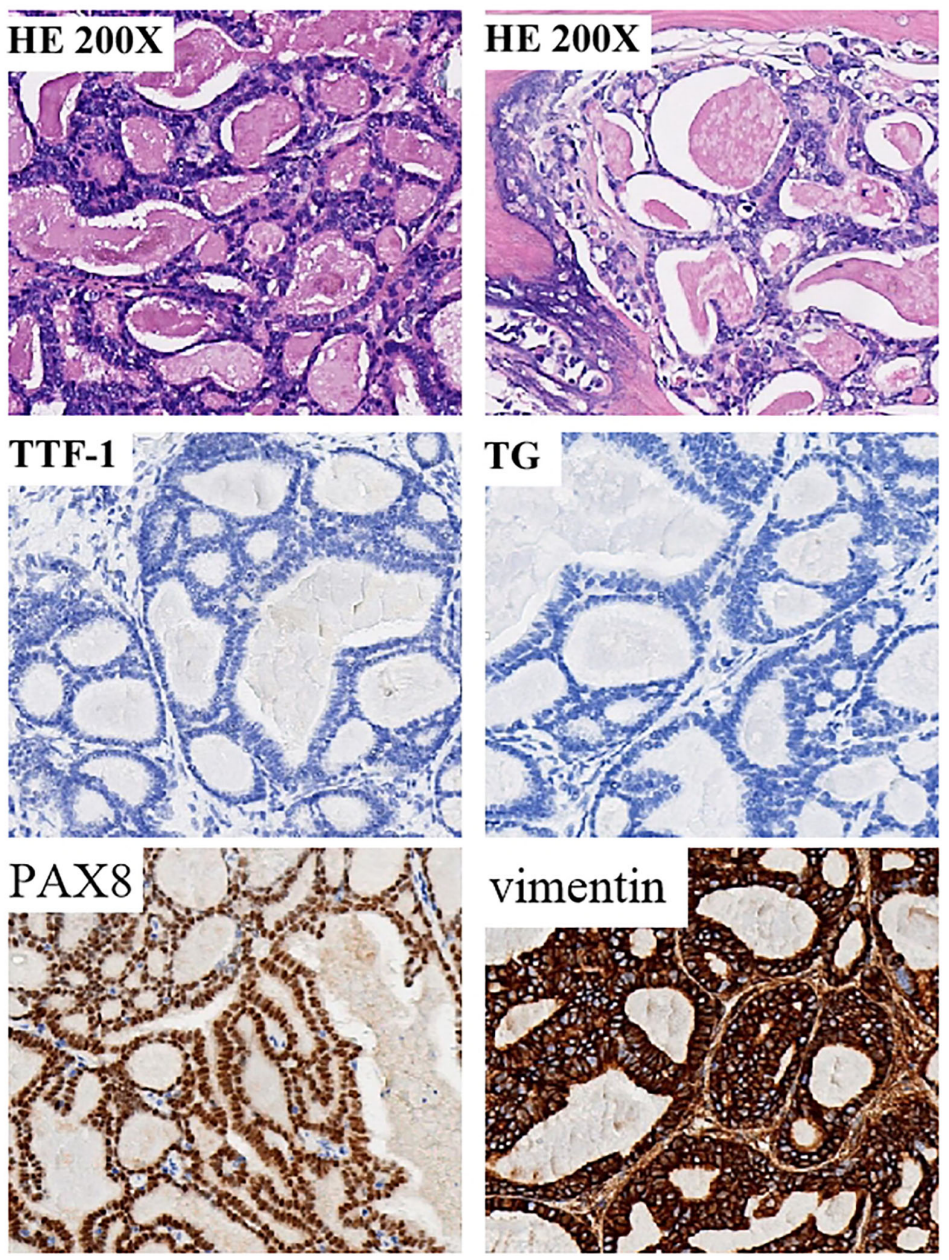
Tumor specimen exhibiting histopathological characteristics consistent with thyroid follicular-like renal cell carcinoma (TLFRCC). Neoplastic cells displayed an assortment of thyroid follicular-like structures varying in size. The presence of fibrosis at the tumor core was accompanied by hyaline degeneration and localized ossification, as evidenced by histological examination (hematoxylin-eosin staining). Immunohistochemical (IHC) analysis revealed distinctive features of thyroid follicular renal cell carcinoma, characterized by positive expression of PAX8 and Vimentin, while the thyroid-specific markers thyroglobulin (TG) and thyroid transcription factor-1 (TTF-1) remained negative.

A 55-year-old man, was first admitted to Wuhan Bauhinia Hospital due to “painless gross hematuria for 3 months” and underwent “laparoscopic radical resection of right renal carcinoma “ under general anesthesia on July 25, 2020 (**Figure 1**). The postoperative pathological diagnosis was TLFRCC (4.2 x 4.0 x3.5cm). It was initially revealed by Wuhan Asian Heart Hospital and verified by our entire pathology department through pathology consultation on July 5, 2023 (**Figure 2**). The patient received sunitinib targeted therapy at one-month intervals, and underwent irregular follow-up reviews after the operation.

**Figure 3:**
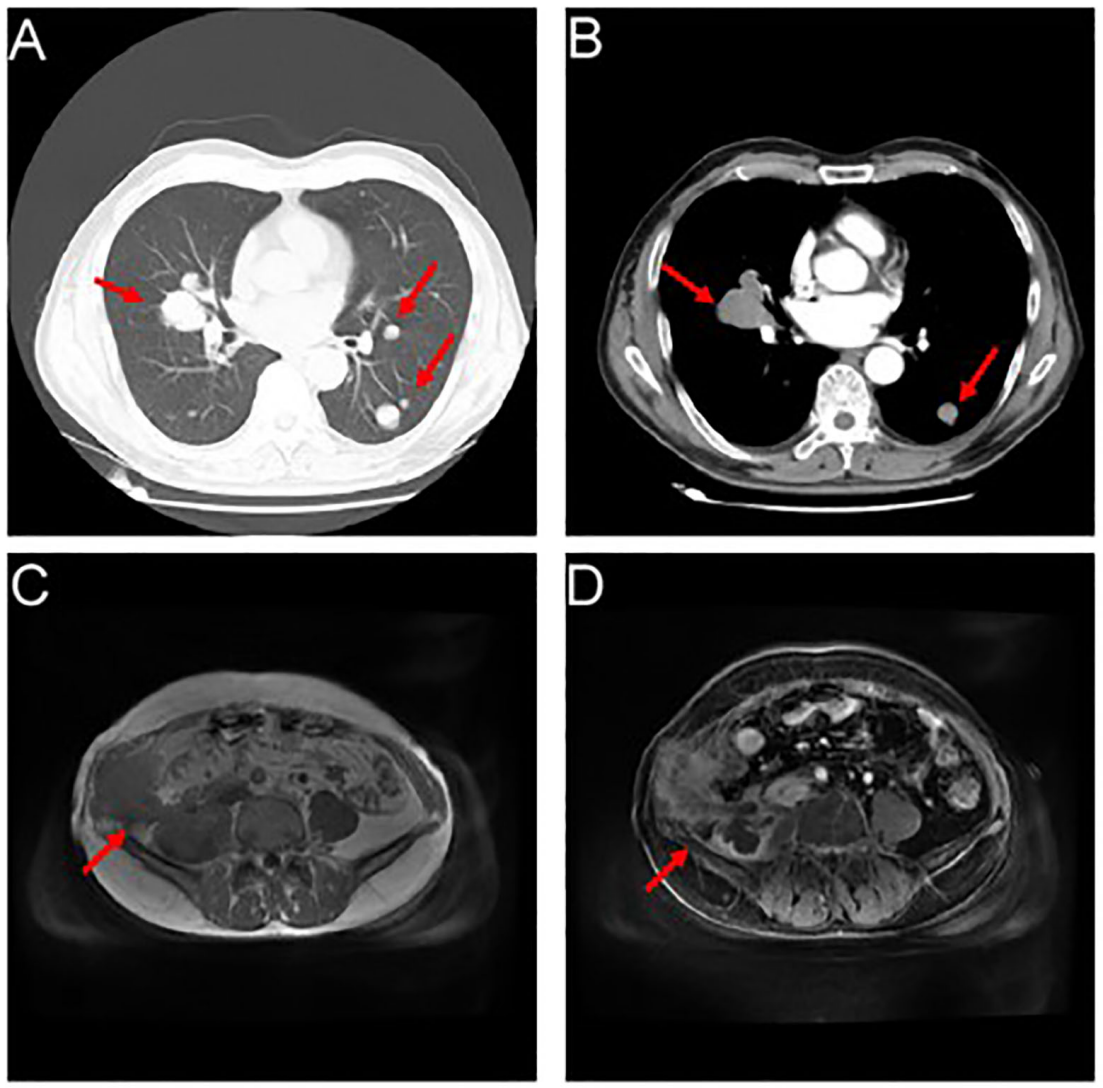
Showcases the occurrence of relapse, accompanied by a proliferation of lung metastatic nodules. **(A, B)** Myriads of nodules (marked by the red arrows), varying in size, emerged within the pulmonary expanse, with a conspicuous prominence residing within the mid-lobe of the dexter lung, proximate to the hilus. The cross-sectional dimensions of this remarkable entity measured approximately 3.7 x 4.1cm. **(C, D)** An assortment of inconstant masses (marked by the red arrows) materialized alongside the right abdominal wall and the formidable psoas major muscle.

**Figure 4:**
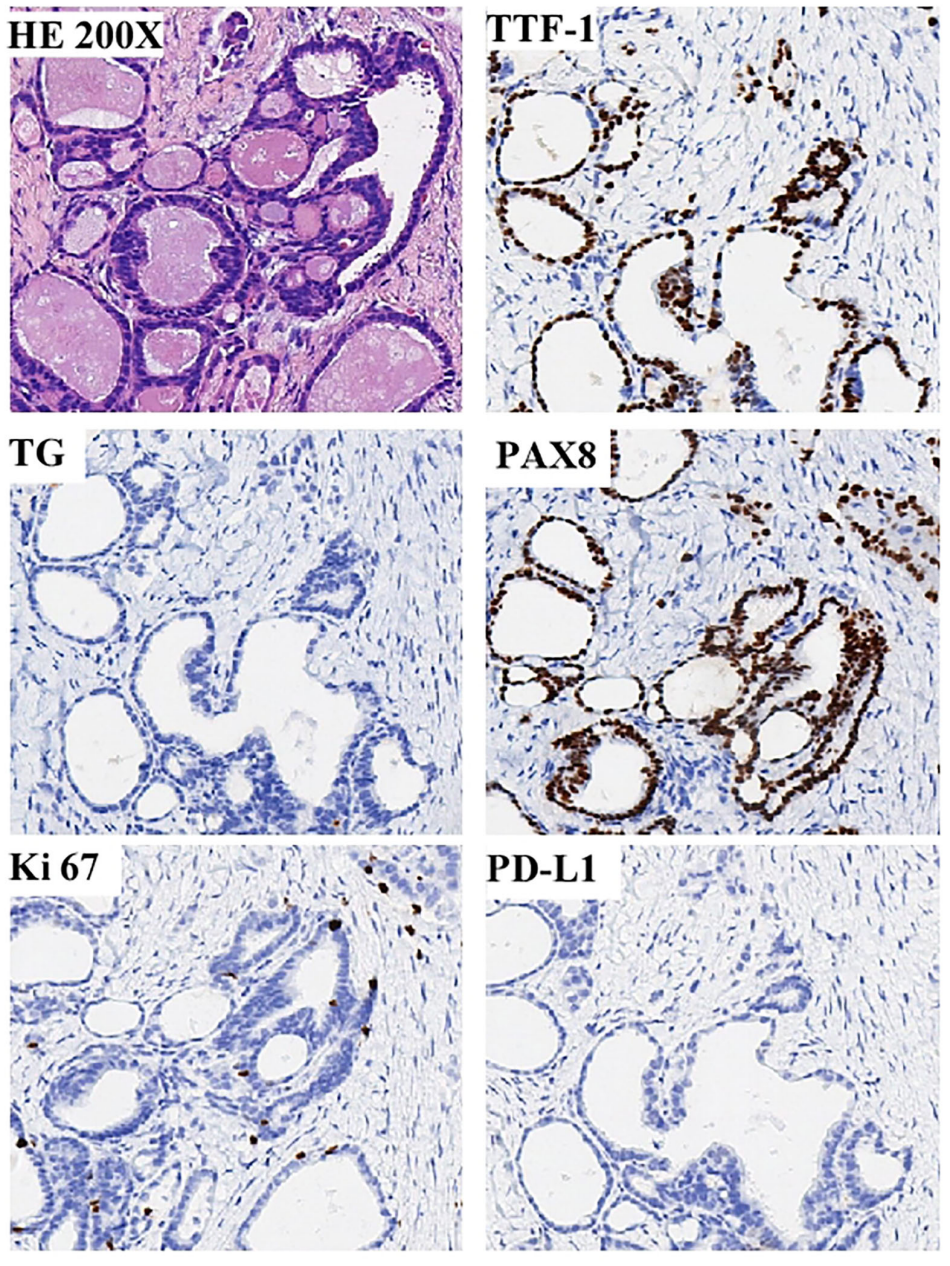
Showcases the compelling pathological discoveries derived from the percutaneous biopsy of the enigmatic pelvic wall mass. A positive expression of PAX8, but negative expression for the thyroid-specific markers thyroglobulin (TG) and thyroid transcription factor-1(TTF-1), and the expression of Ki-67 proliferation index was low, about 5%, and the PD-L1 was negative.

The patient developed right lower abdominal pain on June 28, 2023, and then was admitted to our hospital for further treatment. During the physical examination, a completely immobile lump, measuring 5.0 x 3.5 x 1.0cm and as hard as bone, was detected. CT and MRI imaging (**Figure 3**) revealed abdominal recurrence and additional metastasis to both lungs. In consideration of the growth characteristics, such as inertia, low Ki-67 proliferation index and rarely metastases of this malignant tumor (2), a puncture biopsy was conducted on the right pelvic wall mass, which verified the metastasis of thyroid-follicular renal cell carcinoma. Additionally, PD-L1 (programmed death ligand-1) was found to be negative (**Table 1**; **Figure 4**).

**Table 1:** Immunohistochemical findings of surgical and puncture specimens.

	TG	TTF-1	PAX8	vimentin	CK7	P504S	Ki67	PD-L1	EMA	CAIX	CD10	CD117
Surgical specimens	(-)	(-)	(+++)	(+++)	(+-)	(-)	5%+	/	/	(+--)	(-)	(-)
Puncture specimens	(-)	(++-)	(+++)	(+++)	(+-)	(-)	5%+	(-)	(±)	(+--)	(-)	(-)

**Figure 5:**
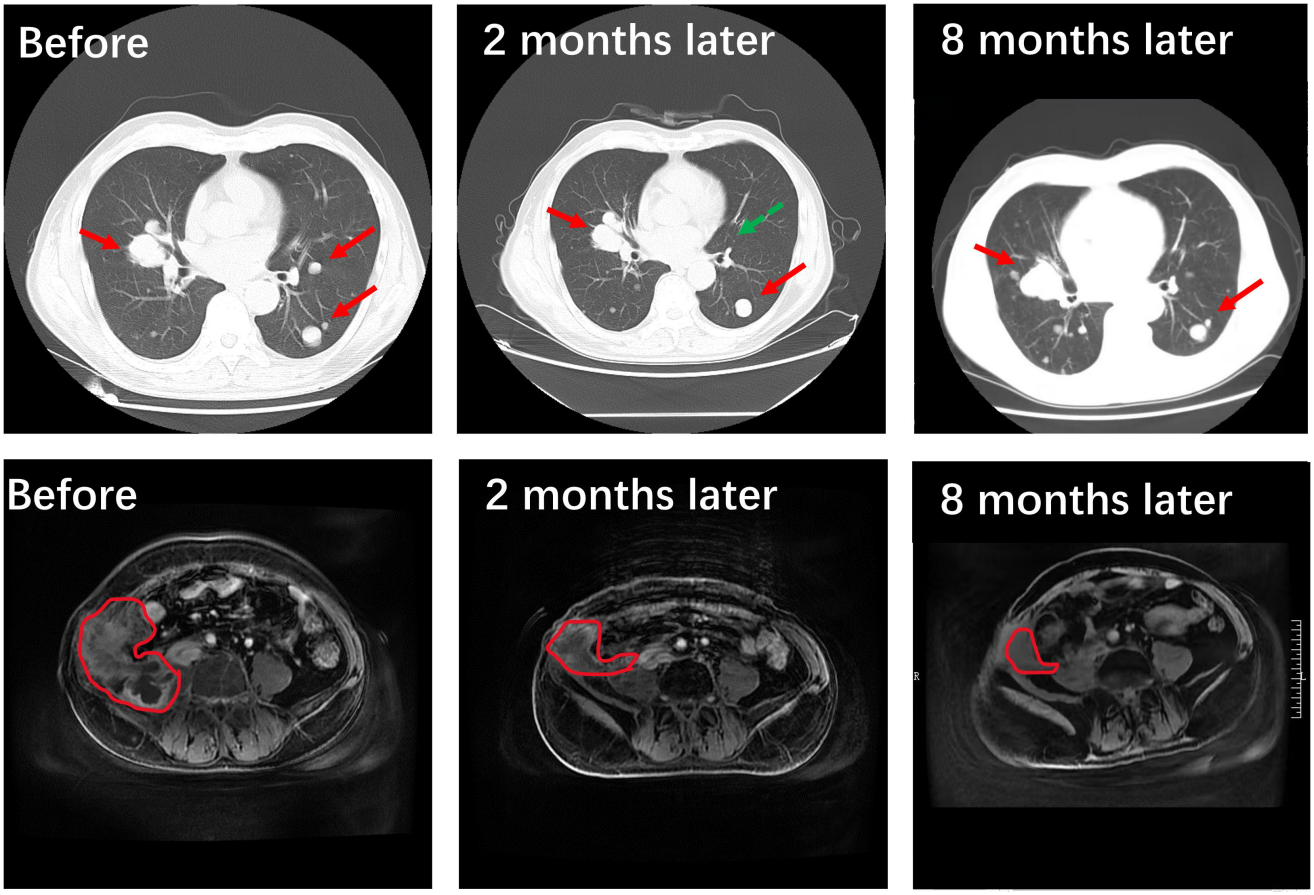
Treatment outcome evaluated by imaging. Compared to the pre-radiotherapy and systemic treatment phase involving a combination of pazopanib and the PD-1 checkpoint inhibitor toripalimab, it is evident through the utilization of advanced imaging techniques such as CT scans and MRI studies that a significant reduction in tumor size (the area outlined by red line) and quantity (the green arrow indicates that the metastatic tumor is almost gone) has been achieved within the pulmonary and abdominal pelvic regions. After 8 months later, the lesions on the lung progressed due to irregular toriplimab treatment, but the lump in the abdomen was still shrinking.

Due to the rarity of the disease and the lack of treatment for metastatic cases, we adopted Illumina high-throughput gene detection technology for sequencing, which combines the latest guideline recommendations and research status. The detection range includes insertion, deletion, point mutation, fusion, CNV (Copy-Number Variants), and other gene variants. As a result, 6 genetic variants were detected, 3 of which have potentially beneficial drugs. Microsatellite instability (MSI) testing via polymerase chain reaction analysis showed that the recurrent tumor was MSS. The mutations of *ATM, POLE, TERT*, and *ERBB2* were found, using a CLIA-certified 30 genes associated with targeted drugs (**Supplementary Table**) and 27 genes related to chemotherapeutic agents at the Clinical Molecular Diagnostics Laboratory, City of Hope National Medical Center, Duarte, CA. The patient was subsequently treated with abdominal radiotherapy (55 Gy in 25 fractions), pazopanib (taken orally, 800 mg/day), and the PD-1 checkpoint inhibitor toripalimab(240mg, add it in 100ml 0.9%Nacl for intravenous infusion). After a few sessions of radiotherapy, the patient’s pain had disappeared. And two months later, the masses in the abdomen and lungs had both shrunk, as assessed by imaging studies (**Figure 5**), after radiotherapy and 2 courses of the above systemic treatment strategies. Which all suggest that TLFRCC cells may be sensitive to radiation. Unfortunately, due to the patient’s personal reasons, irregular toriplimab treatment was carried out about 3 months every time, and the lesions on the lung progressed, but the lump in the abdomen was still shrinking after 8 months (**Figure 5**). The patient is alive 9 months at least, and the follow up will be continued.

## 3 Discussion and conclusion

As mentioned before, TLFRCC was under the category of “other renal tumors”, which includes a group of diverse, unrelated renal tumors that do not fit into other categories in The World Health Organization (WHO) 2022 classification of urinary and male genital tumors (5th edition). TLFRCC is predominantly found in women and can occur across a wide age range, from 10 to 83 years (6, 12). Previous reports have shown that TLFRCC progresses slowly, and most patients do not exhibit obvious clinical symptoms. Instead, the disease is often discovered accidentally during physical examinations (4).

The precise molecular abnormality in TLFRCC is not entirely established; however, it has been acknowledged that the fusion of EWSR1::PATZ1, as a recurrent alteration in a subset of sarcomas exhibiting round to spindle cell morphology and harboring EWSR1–non-ETS fusions (referred to as EWSR1::PATZ1 sarcoma), is also found in multiple central nervous system tumors. This discovery of the EWSR1::PATZ1 fusion in three TLFRCC patients, whose adequate tissue was available for genomic analysis, provides the initial evidence supporting its classification as a distinct diagnostic entity (13). Based on recently cited references, there have been reports of aggressive clinical behavior in cases of TLFRCC characterized by extensive sarcomatous differentiation (14).

This study integrates the most up-to-date recommendations from guidelines and research findings, utilizing Illumina high-throughput gene detection technology for sequencing. The scope of detection includes various gene variants, such as insertions, deletions, point mutations, fusions, and copy-number variants (CNVs). Consequently, six genetic variants were identified, three of which exhibit potential responsiveness to beneficial therapeutic agents (**Table 2**).

**Table 2:** Tumor molecular profiles of known or potential significance.

Gene	Positive Tests	Chr	Nucleotide Change	Mutation Frequency	Potentially Beneficial Drugs
*ATM*	ex on7 c.832delG	11	p.V278Sfs*42	3.20%	Olapari, talazoparil, Ceralasertib + Olaparib, averumab + talazoparil, Nilaparib, Rucaparib, PD-1/PD-L1 inhibitors
*POLE*	ex on40 c.5387dupA	12	p.S1797Efs*38	2%	PD-1/PD-L1 inhibitors
*TERT*	C228T	/		16%	INO-1400, INO-9012, INO-1401 (NCTO2960594)
*ATM*	c.8762C>T	11	p.T2921M	46.70%	/
*POLE*	c.4322T>A	12	p.L1447Q	3.36%	/
*ERBB2*	c.1200T>G	17	p.F400L	1.82%	/

The *ATM* gene, known as a suppressor of tumor growth, is situated on chromosome 11. Its encoded protein belongs to the PI3/PI4 kinase family and serves as a crucial phosphorylated kinase involved in the regulation of the cell cycle checkpoint. In response to DNA damage, the *ATM* gene initiates phosphorylation, thereby activating the expression of downstream target genes responsible for repairing DNA. This repair mechanism works by slowing down the progression of the cell cycle. In cases where the repair process becomes challenging, the *ATM* gene triggers the phosphorylation of MDM2 and p53 proteins, effectively pausing the cell cycle or inducing prolonged apoptosis. As a consequence, the *ATM* gene prevents the degeneration of cells. The inactivation of the *ATM* gene results in a loss of cell repair function, leading to the deterioration of cancerous cells. However, this loss of repair function also renders cancerous cells more susceptible to the effects of radiotherapy. Mutations in the *ATM* gene have been identified in breast cancer, lung cancer, and various lymphatic hematopoietic tumors (15–17). This particular patient harbors the V278Sfs*42 mutation within the *ATM* gene, characterized as a frameshift mutation. Such an alteration holds the potential to prematurely curtail protein coding, culminating in the production of abridged protein entities that can inflict considerable impact on protein functionality.

POLE and POLD1 exemplify the exquisite role of DNA proofreading enzymes, wherein mutations residing within the exonuclease domain, specifically at key residues P286, V411, and S459 of POLE, confer a predisposition for alarmingly elevated rates of base substitution mutations. Clinical investigations have substantiated that patients harboring *POLE* mutations in solid tumors may find solace in therapeutic interventions encompassing PD-1/PDL1 inhibitors, thus reaping potential benefits (18–21). The patient achieved a significant therapeutic response, notwithstanding the negative expression of PD-L1. This favorable outcome can likely be attributed to the presence of *POLE* mutation or the administration of pazopanib, which specifically targets *ATM*.

According to reports, localized cases of TLFRCC that have obtained a prolonged disease-free period following resection of the primary tumor necessitate primarily surgical intervention as the predominant modality to address this malignancy. Whether through radical or partial excision, surgical management stands as the foremost approach for treating this malignant neoplasm (2). Unfortunately, despite the patient’s favorable response to a cytoreductive radical nephrectomy accompanied by an extensive retroperitoneal lymph node dissection (including paracaval, retrocaval, and interaortocaval regions), their subsequent survival spanned a mere three months. This distressing outcome was a consequence of the presence of TLFRCC metastases within the lungs and retroperitoneal lymph nodes (3). Another one, with pelvic and spine bone metastases, received sunitinib and was stable for 6 months, but didn’t respond to nivolumab, and transitioned to best supportive care (22). Our case has further elucidated the potentially belligerent and metastatic nature of TLFRCC, while simultaneously presenting a detailed exposition of groundbreaking systemic therapies employed in the pursuit of treating metastatic TLFRCC. As of present, the patient has exhibited weight gain and maintained a commendable health status at least 9 months. Progression-free survival may be longer if patients are treated with standard toripalimab and continuous pazopanib. Moreover, the regression of abdominal metastases surpasses that of pulmonary metastases, suggestive of the efficaciousness of radiotherapy. To summarize, our report proffers a novel outlook on TLFRCC. However, the question of whether thyroid follicular renal carcinoma evinces sensitivity towards chemotherapeutic agents remains unaddressed. Evidently, further investigation is indispensable to unravel the intricacies of TLFRCC, and a deeper exploration of treatments is imperative.

## Data availability statement

The datasets presented in this study can be found in online repositories. The names of the repository/repositories and accession number(s) can be found in the article/**Supplementary Material**.

## Ethics statement

The studies involving humans were approved by Ethics Committee of Renmin Hospital of Wuhan University. The studies were conducted in accordance with the local legislation and institutional requirements. The participants provided their written informed consent to participate in this study. The manuscript presents research on animals that do not require ethical approval for their study. Written informed consent was obtained from the individual(s) for the publication of any potentially identifiable images or data included in this article.

## Author contributions

J-JL: Writing – original draft, Formal analysis, Software, Visualization. JR: Data curation, Writing – original draft. H-YF: Data curation, Writing – original draft. D-DC: Conceptualization, Writing – review & editing. H-LY: Methodology, Writing – review & editing. J-PY: Validation, Writing – review & editing. Z-MJ: Project administration, Writing – review & editing. Y-QZ: Resources, Supervision, Writing – review & editing.
